# A new theoretical approach to determine the air outlet temperature of an air-to-ground heat exchanger

**DOI:** 10.1016/j.mex.2024.102837

**Published:** 2024-07-02

**Authors:** Marc Sainclair Sokom Efanden, Flavian Emmanuel Sapnken, Benjamin Salomon Diboma, Aubin Kinfack Jeutsa, Jean Gaston Tamba

**Affiliations:** a Laboratory of Technologies and Applied Science, PO Box 8698, IUT Douala, Douala, Cameroon; b Transports and Applied Logistics Laboratory, University Institute of Technology, University of Douala, PO Box 8698 Douala, Cameroon; c Energy Insight-Tomorrow Today, PO Box 2043 Douala, Cameroon; d Higher Technical Teachers' Training College, University of Buea, PO Box 63, Buea, Cameroon

**Keywords:** Air-to-ground heat exchanger, Control volume, Temperature, Equatorial zone, Finite element method for the control volume

## Abstract

In this study, the control volume method is used to determine the air temperature at the outlet of an air-to-ground heat exchanger. Its implementation consists in dividing the duct of the ground-air heat exchanger into micro-volumes of identical size. An energy balance is then established for each micro-volume. The input parameters used to implement this model are related to the city of Yaoundé in the equatorial zone. The results show that when the total length of the air-to-ground heat exchanger duct varies between 0 and 100 m, the air temperature at the outlet also varies between 34.5 and 24 °C. The air-to-ground heat exchanger operates in cooling mode. As the length of the air-to-ground heat exchanger duct increases, the temperature of the air at the outlet of the air-to-ground heat exchanger decreases, approaching that of the ground. Based on the results obtained using the control volume model, the minimum total length of air-to-ground heat exchanger duct recommended for this zone is 40 m. Admittedly, air pressure drops, air humidity and the geometry of the air-to-ground heat exchanger are aspects that have not yet been taken into account in the implementation of this model. Nevertheless, the control volume method can be used to optimise the parameters influencing the thermal performance of an air-to-ground heat exchanger.

• The control volume method is implemented here by dividing the air-to-ground heat exchanger duct into identical micro-volumes and then establishing an energy balance for each micro-volume;
• In this work, the control volume method was used to optimise the total length of the duct of a ground air heat exchanger installed in an equatorial zone;
• Some important aspects such as air pressure drops, air humidity, and the geometry of the air-to-ground heat exchanger are not yet taken into account in the implementation of the control volume method.

## Graphical abstract

**Figure:**
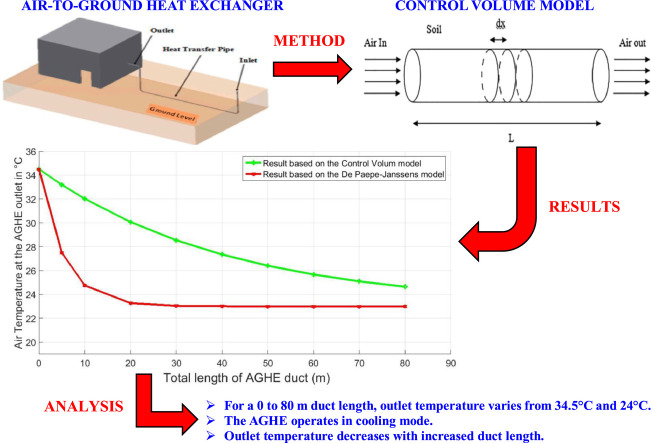

## Nomenclature

A: area (m^2^)
D_i_: inside diameter of EAHE duct (m)
D_o_: external diameter of EAHE duct (m)
f: fractional losses coefficient
k: thermal conductivity (W/m.K)
$\overset{˙}{m}$: mass flow rate (kg.s^-1^)
Nu: number of Nusselt
*C*: specific heat of air (J.kg^-1^.K^-1^)
$h_{c}$: duct heat transfer coefficient (J/m K^2^)
*P*: perimeter (m)
Pr: number of Prandlt
Re: Reynolds number
*t*: time (hours)
*Tc*: EAHE duct temperature ( °C or K)
*Te*: air inlet temperature into EAHE duct ( °C or K)
*To*: EAHE duct air outlet temperature ( °C or K)
*Ts*: ground temperature ( °C or K)
*V_a_*: air speed (m.s^-1^)
*U*: overall heat transfer coefficient (J/m.K^2^)
*w*: moisture ratio (kg/kg)

**Greek symbols**
*β*: convective mass transfer coefficient (m/s)
*ρ*: density (kg.m^3^)
*µ*: dynamic viscosity (Kg.m^-1^.s^-1^)
*ω*: diffusion coefficient

**Acronyms**
EAHE: earth-air heat exchanger
AGHE: air-to-ground heat exchanger

Specifications table

**Table:** 

Subject area:	Energy
More specific subject area:	Surface geothermal energy
Name of your method:	*Finite element method for the control volume*
Name and reference of original method:	M. Sheikholeslami, Chapter 1 - Detailed Explanation of Control Volume-based Finite Element Method, in: M. Sheikholeslami (Ed.), Appl. Control Vol. Based Finite Elem. Method CVFEM Nanofluid Flow Heat Transf., Elsevier, 2019: pp. 1–13. 10.1016/B978–0–12–814,152–6.00001–1. M. Kaushal, Performance analysis of clean energy using geothermal earth to air heat exchanger (GEAHE) in Lower Himalayan Region - Case study scenario, Energy Build. 248 (2021) 111,166. 10.1016/j.enbuild.2021.111166.
Resource availability:	National climate change observatory (http://www.onacc.cm/) weather-history.net (https://www.historique-meteo.net)

### Background

The finite volume method and the finite element method are combined to create the control volume method, which incorporates interesting features of both approaches [1,2]. This analytical method has been developed in various ways with the aim of solving scientific problems of various kinds. This methodology, for instance, has been applied to the 3D prediction of radiative heat flow in complicated geometries and their constrained surroundings [3,4]. It has also made it possible to solve the problem of non-axisymmetric radiative transfer in inhomogeneous cylindrical media that are anisotropic emitters, absorbers and even scatters [5]. The coupled heat and mass transfer that occurs in a metal-hydrogen reactor equipped with a phase-change aterial has been predicted by developping a transient two-dimensional mathematical model. Mâad et al. [6] used the control volume method to discretise the basic equations. Mello et al. [7] solved the coupled partial differential equations governing geological processes in sedimentary basin evolution using the 3D control volume method. Shojaei et al. utilized the control volume method to predict flow progression, pressure distribution, and mould clamping force during compression resin transfer moulding [8]. Turner and Ferguson [9] analysed the control volume method centred on unstructured mesh cells and then applied it to high-temperature drying of softwood. Zhang et al. [10] employed the control volume method to study fluid flow dynamics in fractured reservoirs. Their research and numerical results provided theoretical guidance for the efficient, large-scale development of natural fractured reservoirs. Beveridge et al. [11] developed a finite element linear strain control volume to predict the effects of micropolar elastic size of materials with obvious internal structure. Their aim was to account for the unconventional behaviour of these types of materials. The developed control volume method successfully passed a micropolar patch test and demonstrated accuracy comparable to its finite element equivalent. An expansion of the control volume method for time-dependent processes in hydraulic networks was created by Skibin and Volkov [12]. In order to compute vertical and guiding forces in high-temperature superconductors while taking flow creep phenomena into account, Alloui et al. [13] developed a 3D computational model. The control volume method's experiments validated its results, but numerical approximation of three-dimensional poroelastic wave equations in spherical coordinate system faces challenges due to a singular problem at the center and polar axes. To overcome this, Zhang et al. [14] developed a hybrid finite difference and control volume method.

The method's convergence rate and effectiveness in simulating wave propagation in poroelastic media in the spherical coordinate system were confirmed. Mohammed et al. [15] developed the control volume method for solving the advection-diffusion equation to solve heat transfer by natural convection in laminar flow. They found that this method gives very similar results to those obtained with other methods. Cruz et al. [16] found that the control volume finite element method is 33 % more accurate than the finite difference method for solving differential equations.

As part of the study of the performance of an air-to-ground heat exchanger (AGHE), the finite element method of the control volume is used to establish an energy balance for each micro-volume of the air-to-ground heat exchanger (AGHE) duct. Mass transfer is therefore taken into account when analysing the performance of an air-to-ground heat exchanger (AGHE). The progression of heat transfer between the air and the ground is thus divided into two parts: latent heat transfer and sensible heat transfer. This represents an evolution insofar as for certain analytical models of air-to-ground heat exchanger performance studies, the total heat transfer is determined by considering exclusively the convective heat transfer of the air circulating in the air-to-ground heat exchanger duct.

The control volume method is flexible, allowing for a balance between accuracy and calculation requirements [17]. In this work, we propose to use an analytical model, by applying it to the determination of the thermal performance of an air-to-ground heat exchanger (AGHE). The aim of this work is to use the analytical method in order to estimate the air temperature at the outlet of an AGHE. The aim is to vary the total length of the AGHE duct, and to observe the effect it has on the thermal behaviour of the air at the AGHE outlet. Based on the results obtained, the length of the AGHE duct can be optimised, taking into account the thermal comfort range of the area where this passive heating and cooling technology is installed.

The contributions of the study can be summarized as follows:

• **Implementation of control volume method:** The study contributes by implementing the control volume method to determine the air temperature at the outlet of an air-to-ground heat exchanger. This involves dividing the duct of the heat exchanger into identical micro-volumes and establishing an energy balance for each micro-volume.
• **Optimization of duct length:** The method optimizes the total length of the duct of a ground air heat exchanger installed in an equatorial zone using the control volume method.
• **Recommendation for minimum duct length:** Based on the results obtained, the study recommends a minimum total length for the air-to-ground heat exchanger duct in the equatorial zone to achieve optimal thermal performance.
• **Potential for further optimization:** Although certain aspects such as air pressure drops, air humidity, and the geometry of the heat exchanger are not yet considered in the model, the study highlights the potential of the control volume method for optimizing parameters influencing the thermal performance of air-to-ground heat exchangers.

Overall, the contributions of the study lie in the application of the control volume method to optimize the duct length of air-to-ground heat exchangers in equatorial zones and in identifying areas for further refinement and optimization in future research.

### Method details

#### Basic assumptions and boundary conditions

In order to better develop the model, certain basic assumptions are considered, namely

(i) The AGHE has a uniform cross-section and uniform thermal characteristics, as the duct is made of a homogeneous material, PVC (Polyvinyl chloride);
(ii) The soil is isotropic and made of a single material;
(iii) There is perfect contact between the AGHE pipe and the soil; in fact, the pipe is buried in the soil, and contact with the soil is direct;
(iv) The thickness of the AGHE pipe has a negligible thermal resistance compared with that of the soil, the thickness of the soil being infinite [18];
(v) The air is characterised by incompression, as its velocity is considered constant during its journey through the AGHE duct;
(vi) The air inside the AGHE duct is homogeneous, as it retains the same thermal properties during its path, and there is no temperature stratification.

The boundary conditions used to implement the model are as follows:

• At the inlet to the air-to-ground heat exchanger, turbulence is average with a constant air flow velocity equal to 1.5 m/s; over a specific time interval, the static temperature at the air inlet varies according to environmental conditions; the thermodynamic properties of the air, such as density, specific heat capacity and thermal conductivity, are defined at the air inlet;
• The AGHE outlet's boundary conditions are equivalent to atmospheric zero, with relative pressure being equal to zero.
• The wall temperature along the surface of the duct is considered to be uniform in the axial direction; this is the undistributed temperature of the soil in the study area; the non-slip condition with the smooth wall is taken into account at the inner surface of the AGHE duct.

#### Study of heat transfer in the AGHE

The air that is transited through the air-ground heat exchanger exchanges heat with the ground, the exchange surface being the duct wall. Implementing the control volume method, the duct (duct + distributed soil) is subdivided into j (in our case, j=8) fictitious layers of length Δx according to the orientation of the air flow, and the energy balance of the layer is established as follows:

The energy balance of the layer for the air inside the duct is given by Eq. (1) [[18], [19], [20]]:

(1) $$\overset{˙}{m}C_{air}\frac{dT_{air,out}^{\left(j\right)}}{dx}=-\left(\pi D_{i}\right)\times U\left(T_{air,in}^{\left(j\right)}-T_{soil}\right)$$

$T_{air,in}^{\left(j\right)}$is the air temperature at the entrance to layer j (°C); $T_{air,out}^{\left(j\right)}$ is the air temperature at the exit of layer j ( °C) . The soil temperature $T_{soil}$ is considered uniform over the entire surface of the distributed soil surrounding the AGHE duct, of internal diameter $D_{i}$ and external diameter $D_{0}$. U is the overall heat transfer coefficient. $\overset{˙}{m}$ is the air mass flow rate ($kg.s^{-1}$), and $C_{air}$ is the specific heat of the air ($J.kg^{-1}.K^{-1})$.

The correlation of the convective heat transfer coefficient between the circulating air and the AGHE duct is given by Eqs. (2)–(6) [21]:

(2) $$h=\frac{k_{air}.Nu}{D_{i}}$$

With:

(3) $$Re=\frac{\rho_{air}.V_{air}.D_{i}}{\mu_{air}}$$

(4) $$Pr=\frac{\mu_{air}.C_{air}}{k_{air}}$$

(5) $$Nu=0.0214.\left(Re^{0.8}-100\right)Pr^{0.4}$$

(6) $$\overset{˙}{m}=\rho_{air}.A.V_{air}\;$$

$k_{air}$ is the thermal conductivity of air ($W.m^{-1}.K^{-1})$;; Nu is the Nusselt number; Re is the Reynolds number; $\rho_{air}$ is the air density $\left(kg.m^{-3}\right)$; $V_{air}$ is the air velocity $\left(m.s^{-1}\right)$; Pr is the Prandlt number; A is the lateral area of the AGHE duct $\left(m^{2}\right)$; $\mu_{air}$ is the dynamic viscosity of the air $\left(kg.m^{-1}.s^{-1}\right)$.

The overall heat transfer coefficient is determined using Eq. (7) [18,19]:

(7) $$U=\frac{1}{\frac{1}{h}+\frac{D_{i}\ln\left(\left(D_{i}+D_{o}\right)\;/\;2D_{i}\right)}{2k_{c}}}$$

By solving Eq. (1) analytically, we can establish the desired output temperature for layer j expressed by Eq. (8) [18]:

(8) $$T_{air,out}^{j}=T_{soil}+\left(T_{air,in}^{j}-T_{soil}\right)exp\left(-\frac{\pi .D_{i}.U.\Delta x}{\overset{˙}{m}.C_{air}}\right)$$

The air temperatures at the outlet of the different layers of the duct are determined successively until the last one is reached, which corresponds to the air temperature at the outlet of AGHE. An internal code using the Matlab language is used to run the model.

### Method validation

In this work, the control volume method is used to determine the air temperature at the outlet of the AGHE. This provides us with the necessary information on the AGHE parameters to be optimised for a better experimentation of this passive building heating and cooling technology. In our case, we have chosen to vary the total length of the AGHE duct in order to analyse its impact on the air temperature at its outlet.

#### Implementation parameters

In order to implement our model, data from the literature are used. To reference the control volume model, the De Paepe-Janssens’ model [22] is used. The De Paepe-Janssens’ model is a one-dimensional analytical methodology for defining the characteristic dimensions of an air-to-ground heat exchanger. The aim is to obtain maximum thermal efficiency from the air-to-ground heat exchanger, while minimising the pressure drop [21]. The different results obtained from these two models will be compared, followed by a brief discussion.

The characteristics of the air and soil are related to the Yaoundé area in Cameroon, which has an equatorial climate. The AGHE has a PVC duct whose total length varies between 0 and 100 m. What interests us here is the air temperature at the outlet of the AGHE. It is installed in lateritic soil at a depth of 3.5 m. At this depth, the soil temperature is constant throughout the year, and is hardly affected by external radiation [23]. The air inlet temperature is 34.5 °C. The floor temperature is constant at 23 °C. The air flows through the air-to-ground heat exchanger duct at a constant speed of 1.5 m/s. There is no pressure drop in the air-to-ground heat exchanger. The thermal properties of the materials used are shown in Table 1, while Table 2 reports the AGHE parameters.

**Table 1:** Thermal properties of the materials used.

Materials	Density ρ (Kg/m)^3^	Specific heat C (J/ Kg. K)	Thermal conductivity λ or k (W/m. K)	Thermal diffusivity a (m /days)^2^	Dynamic viscosity µ (Kg.m^-1^ . s)^−1^	Refs.
Air	1.204	1006	0.0257	200	1.81 × 10^–5^	[24]
Soil	1500	–	1.7	–	–	[25]
PVC	1380	960	0.17	0.01547		[26]

**Table 2:** AGHE parameters.

Parameters	Reference values
Conduit length	(0–80) m
Inside diameter of duct	80 mm
Duct thickness	5 mm
Air flow rate	1.5 m/s
Ground temperature	23 °C
AGHE air inlet temperature	34.5 °C

In our case, the air-to-ground heat exchanger is assumed to be installed at a depth of 3.5 m in the ground. At this depth, the ground temperature is constant and is hardly affected by external radiation [23].

#### Simulation results

**Fig. 1:**
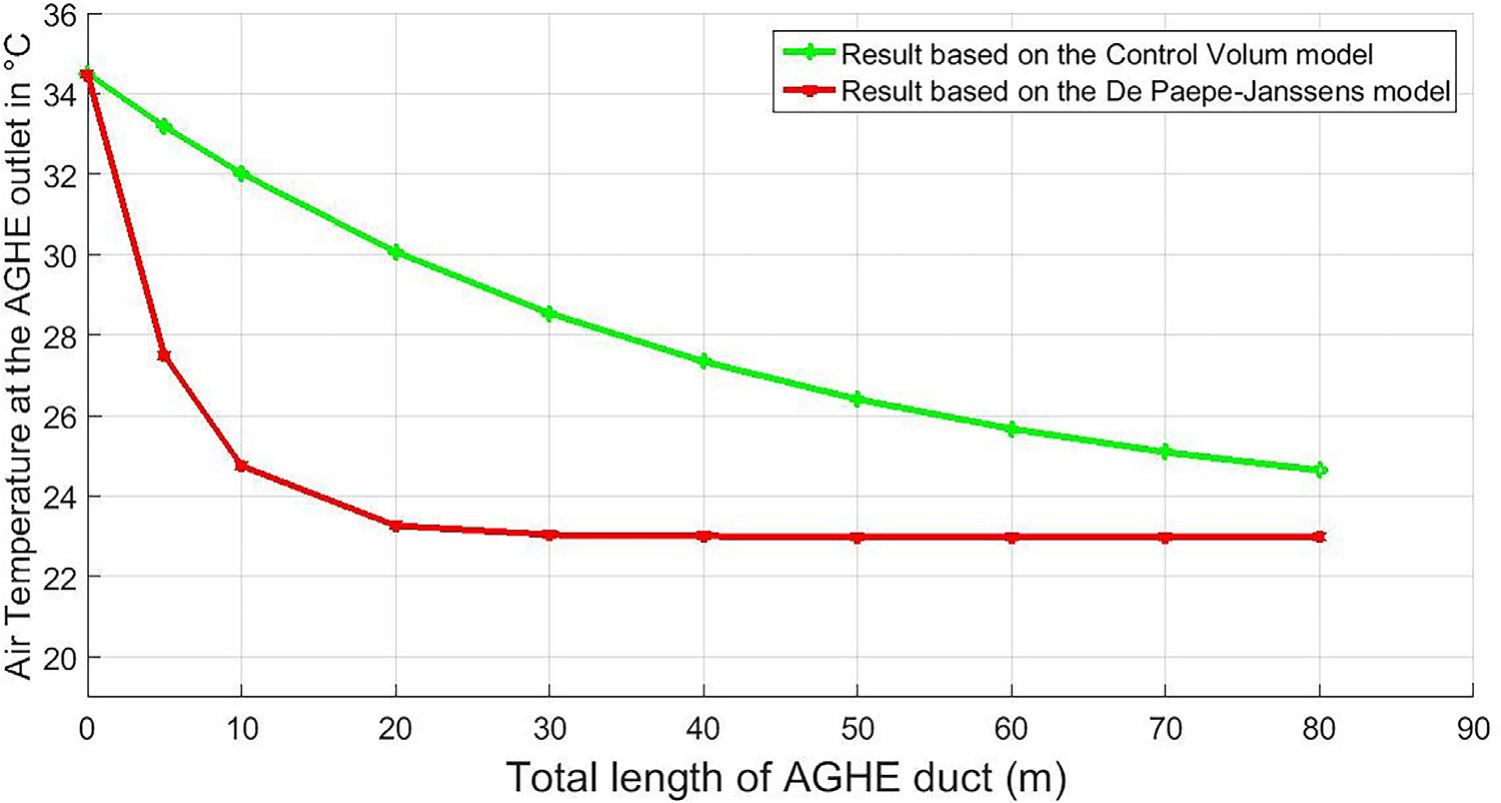
Air temperature variation as a function of the length of the AGHE duct.

Fig. 1 shows two curves. The green curve represents the evolution of the air outlet temperature of AGHE as a function of its total duct length according to the control volume model. The green curve represents the evolution of the air outlet temperature of AGHE as a function of its total duct length according to the De Paepe-Janssens model. According to this model, the heat exchanger in question is able to supply air temperatures equal to the ground temperature for a minimum duct length of 30 m. The point of view is different for the control volume model. According to this model, this air-to-ground heat exchanger cannot supply air at a temperature equivalent to that of the ground, even for a maximum total duct length of 80 m. This is more realistic than the De Paepe-Janssens model. In the De Paepe-Janssens model, the total heat transfer is determined by considering only the convective heat transfer of the air circulating in the air-to-ground heat exchanger duct. However, in the control volume model, the progression of heat transfer between the air and the ground is split between latent heat transfer and sensible heat transfer. This makes the results obtained with this model more credible.

Table 3 shows the different temperatures of the air leaving the AGHE obtained according to the models, for the different total lengths of AGHE ducts considered. It shows that the AGHE operates in cooling mode. The temperature of the air leaving the AGHE decreases as the length of the AGHE duct increases. As the length of the AGHE duct increases, the temperature of the air leaving the AGHE decreases and approaches the temperature of the ground. When the total length of the air-to-ground heat exchanger duct exceeds 80 m, the temperature of the air at its outlet varies minimally, its value approaching that of the ground, which is 23 °C.

**Table 3:** Different air temperature results obtained at the AGHE outlet.

Total length of AGHE duct (m)	Air temperature at AGHE outlet ( °C) according to control volume model	Air temperature at AGHE outlet ( °C) according to De Paepe-Janssens model [22]
10	32	24.8
20	30.0	23.3
30	28.5	23.0
40	27.3	23.0
50	26.3	23.0
60	25.6	23.0
70	25.0	23.0
80	24.6	23.0

These results, obtained using the control volume method, are important. In our case, they allow us to determine the length of AGHE ducting required. Depending on the thermal comfort range of the zone where the AGHE is supposed to be installed, these results allow us to specify the length of AGHE ducting needed to provide the desired air temperature(s) in the building. In the case in point, when we consider the climatic conditions in the city of Yaoundé, according to T. Nganya et al. [27] and Mba et al. [28], the thermal comfort temperature range in this zone is between 23.3 and 27.2 °C. Based on the results obtained using the control volume model, the minimum total length of AGHE duct recommended for this zone is 40 m.

However, it should be pointed out that the control volume method needs to be improved insofar as it does not take into account a number of important considerations. These include: the pressure drop of the air as it travels through the AGHE duct [29]; the humidity of the air, which is an important factor, as the air in general undergoes condensation as it travels through the AGHE duct [30,31]; the geometry of the AGHE, which is an important aspect [32,33], and is hardly taken into account in the current formulation of the control volume method. Nevertheless, this in no way detracts from the fact that the control volume method remains an interesting model for the parametrisation of AGHE. Its evolution is therefore a major challenge which will certainly be the subject of our future work.

### Limitations

The method described in this paper has several limitations that may affect its applicability under certain experimental conditions:

• **Assumption validity:** The accuracy of the method relies on assumptions such as uniform micro-volume sizes and steady-state conditions. Deviation from these assumptions, such as non-uniform flow distribution or transient operation, may lead to inaccurate predictions.
• **Spatial and temporal resolution:** The discretization of the heat exchanger into micro-volumes introduces spatial discretization errors, especially if the heat exchanger geometry is complex or irregularly shaped. Additionally, the method assumes steady-state conditions, potentially limiting its applicability for systems with rapid changes in operating conditions.
• **Parameter sensitivity:** The method's accuracy is influenced by parameters such as thermal conductivity, heat capacity, and flow rates, which must be accurately known or estimated. Uncertainties or variations in these parameters may affect the reliability of the predictions.
• **Model complexity:** While the method offers flexibility in balancing accuracy and computational requirements, overly simplified models may not capture complex heat transfer phenomena accurately. As such, the method's applicability may be limited for systems with non-linear or multi-physics behaviour.
• **Experimental validation:** The method's predictive accuracy should ideally be validated against experimental data from real-world air-to-ground heat exchangers. Lack of experimental validation may limit confidence in the method's predictive capability, especially under varying operating conditions or system configurations.

### Conclusion

In this work, the control volume method is used to estimate the air temperature at the outlet of an air-to-ground heat exchanger. The input parameters used to implement this model are related to an equatorial zone. The results show that when the total length of the air-to-ground heat exchanger duct varies between 0 and 80 m, the air temperature at the outlet also varies between 34.5 and 24.6 °C. The air-to-ground heat exchanger operates in cooling mode. Based on the results obtained using the control volume model, the minimum total length of AGHE duct recommended for this zone is 40 m. Admittedly, air pressure drops, air humidity levels and the geometry of the air-to-ground heat exchanger are aspects that have not yet been taken into account in the implementation of this model. Nevertheless, the control volume method is a means of optimising the parameters influencing the thermal performance of an air-to-ground heat exchanger. It only needs to be improved by finding solutions to the shortcomings observed, and by comparing it with the other proven analytical methods found in the literature. All these avenues of research will be explored in future work.

### Ethics statements

The authors of this work have read and complied with the ethical requirements for publication in MethodsX and the current work does not involve human subjects, animal experiments or data collected from social media platforms.

### CRediT authorship contribution statement

**Marc Sainclair Sokom Efanden:** Conceptualization, Methodology, Investigation, Writing – original draft. **Flavian Emmanuel Sapnken:** Visualization, Investigation, Writing – review & editing. **Benjamin Salomon Diboma:** Visualization, Investigation, Writing – review & editing. **Aubin Kinfack Jeutsa:** Software, Data curation, Formal analysis. **Jean Gaston Tamba:** Supervision, Validation, Project administration.

## Declaration of competing interest

The authors declare that they have no known competing financial interests or personal relationships that could have appeared to influence the work reported in this paper.

## Data Availability

Data will be made available on request.

## References

[1] Sheikholeslami M. (2019). Application of Control Volume Based Finite Element Method (CVFEM) For Nanofluid Flow and Heat Transfer.

[2] Kandelousi M.S., Ganji D.D. (2015). Hydrothermal Analysis in Engineering Using Control Volume Finite Element Method.

[3] Salah M.B., Askri F., Rousse D., Nasrallah S.Ben (2005). Control volume finite element method for radiation. J. Quant. Spectrosc. Radiat. Transf..

[4] Grissa H., Askri F., Ben Salah M., Ben Nasrallah S. (2010). Prediction of radiative heat transfer in 3D complex geometries using the unstructured control volume finite element method. J. Quant. Spectrosc. Radiat. Transf..

[5] Grissa H., Askri F., Ben Salah M., Ben Nasrallah S. (2008). Nonaxisymmetric radiative transfer in inhomogeneous cylindrical media with anisotropic scattering. J. Quant. Spectrosc. Radiat. Transf..

[6] Ben Mâad H., Miled A., Askri F., Ben Nasrallah S. (2016). Numerical simulation of absorption-desorption cyclic processes for metal-hydrogen reactor with heat recovery using phase-change material. Appl. Therm. Eng..

[7] Mello U.T., Rodrigues J.R.P., Rossa A.L. (2009). A control-volume finite-element method for three-dimensional multiphase basin modeling. Mar. Pet. Geol..

[8] Shojaei A., Boorboor D., Ghaffarian S.R. (2004). Composite Technologies for 2020.

[9] Turner I.W., Ferguson W.J. (1995). An unstructured mesh cell-centered control volume method for simulating heat and mass transfer in porous media: application to softwood drying, part I: the isotropic model. Appl. Math. Model..

[10] Zhang R., Zhang L., Luo J., Yang Z., Xu M. (2016). Numerical simulation of water flooding in natural fractured reservoirs based on control volume finite element method. J. Pet. Sci. Eng..

[11] Beveridge A.J., Wheel M.A., Nash D.H. (2013). A higher order control volume based finite element method to predict the deformation of heterogeneous materials. Comput. Struct..

[12] Skibin A., Volkov V. (2014). Extending the control−volume method to unsteady network hydraulic simulations. Procedia Eng..

[13] Alloui L., Bouillault F., Bernard L., Lévêque J., Mimoune S.M. (2012). 3D modeling of forces between magnet and HTS in a levitation system using new approach of the control volume method based on an unstructured grid. Phys. C Superconduct..

[14] Zhang W., Tong L., Chung E.T. (2014). A hybrid finite difference/control volume method for the three dimensional poroelastic wave equations in the spherical coordinate system. J. Comput. Appl. Math..

[15] Mohammed H., Belkacem A., Noureddine K., elhadj B. (2017). Control volume finite element method for a benchmark validation of a natural convection in a square cavity. Energy Procedia.

[16] Cruz P., Santos J.C., Magalhães F.D., Mendes A. (2005). Comparison of finite difference and control volume methods for solving differential equations” by G.G. Botte, J.A. Ritter, R.E. White, 24 (2000) 2633–2654. Comput. Chem. Eng..

[17] Ali H.B., Askri F., Nasrallah S.B. (2017). Comparative study of WSGG and SLW models coupled with control volume finite element method for non gray radiation prediction. Int. J. Therm. Sci..

[18] Kaushal M. (2021). Performance analysis of clean energy using geothermal earth to air heat exchanger (GEAHE) in Lower Himalayan Region – Case study scenario. Energy Build..

[19] Benhammou M., Draoui B., Zerrouki M., Marif Y. (2015). Performance analysis of an earth-to-air heat exchanger assisted by a wind tower for passive cooling of buildings in arid and hot climate. Energy Convers. Manage.

[20] Ascione F., Bellia L., Minichiello F. (2011). Earth-to-air heat exchangers for Italian climates. Renew. Energy.

[21] Papakostas K.T., Tsamitros A., Martinopoulos G. (2019). Validation of modified one-dimensional models simulating the thermal behavior of earth-to-air heat exchangers—Comparative analysis of modelling and experimental results. Geothermics.

[22] De Paepe M., Janssens A. (2003). Thermo-hydraulic design of earth-air heat exchangers. Energy Build..

[23] Agrawal K.K., Misra R., Agrawal G.D. (2021). CFD simulation study to evaluate the economic feasibility of backfilling materials for ground-air heat exchanger system. Geothermics.

[24] Adol W., Mvogo P., Ayissi M., Mouangue R. (2021). Experimental and analytical studies on heat transmission inside EAHE in tropical zone. J. Mater. Environ. Sci..

[25] Meukam P., Noumowe A., Jannot Y., Duval R. (2003). Caractérisation thermophysique et mécanique de briques de terre stabilisées en vue de l'isolation thermique de bâtiment. Mat. Struct..

[26] Zhao Y., Li R., Ji C., Huan C., Zhang B., liu L. (2019). Parametric study and design of an earth-air heat exchanger using model experiment for memorial heating and cooling. Appl. Therm. Eng..

[27] Nganya T., Ladevie B., Kemajou A., Mba L. (2012). Elaboration of a bioclimatic house in the humid tropical region: case of the town of Douala-Cameroon. Energy Build..

[28] Mba L., Meukam P., Kemajou A. (2016). Application of artificial neural network for predicting hourly indoor air temperature and relative humidity in modern building in humid region. Energy Build..

[29] Amanowicz Ł. (2018). Influence of geometrical parameters on the flow characteristics of multi-pipe earth-to-air heat exchangers – experimental and CFD investigations. Appl. Energy.

[30] Morshed W., Leso L., Conti L., Rossi G., Simonini S., Barbari M. (2018). Cooling performance of earth-to-air heat exchangers applied to a poultry barn in semi-desert areas of south Iraq. Int. J. Agric. Biol. Eng..

[31] Rosa N., Santos P., Costa J., Gervásio H. (2018). Modelling and performance analysis of an earth-to-air heat exchanger in a pilot installation. J. Build. Phys..

[32] Agrawal K.K., Agrawal G.D., Misra R., Bhardwaj M., Jamuwa D.K. (2018). A review on effect of geometrical, flow and soil properties on the performance of Earth air tunnel heat exchanger. Energy Build..

[33] Agrawal K.K., Misra R., Agrawal G.D., Bhardwaj M., Jamuwa D.K. (2019). Effect of different design aspects of pipe for earth air tunnel heat exchanger system: a state of art. Int. J. Green Energy.

